# In vitro quality evaluation of leading brands of ciprofloxacin tablets available in Bangladesh

**DOI:** 10.1186/s13104-017-2507-y

**Published:** 2017-05-30

**Authors:** Md. Sahab Uddin, Abdullah Al Mamun, Md. Saddam Hossain, Md. Asaduzzaman, Md. Shahid Sarwar, Mamunur Rashid, Oscar Herrera-Calderon

**Affiliations:** 1 Department of Pharmacy, Southeast University, Dhaka, Bangladesh; 2 Department of Pharmacy, Noakhali Science and Technology University, Noakhali, Bangladesh; 3 Department of Pharmacy, University of Rajshahi, Rajshahi, Bangladesh; 4 Academic Department of Pharmaceutical Sciences, Faculty of Pharmacy and Biochemistry, Universidad Nacional San Luis Gonzaga de Ica, Ica, Peru

**Keywords:** Quality control, Ciprofloxacin hydrochloride, Leading brands, Pharmacopoeial specifications

## Abstract

**Background:**

Ciprofloxacin is a broad-spectrum antibiotic that acts against a number of bacterial infections. The study was carried out to examine the in vitro quality control tests for ten leading brands of ciprofloxacin hydrochloride 500 mg tablet formulation, registered in Bangladesh by Directorate General of Drug Administration. The quality control parameters of ten different brands of ciprofloxacin hydrochloride 500 mg tablets were determined by weight variation, friability, hardness, disintegration, dissolution and assay tests. All the tablets were evaluated for conformity with United States Pharmacopoeia-National Formulary (USP-NF) and British Pharmacopoeia (BP) standards.

**Results:**

Among ten brands of tablets Brand C had lower mean weight variation of 1.59% and Brand E had highest mean weight variation of 3.32%. For friability test Brand F had lowest mean friability (0.27%) and Brand G had highest mean friability (0.54%). Among ten brands mean lowest and highest hardness were founded in Brand G (4.49 kg/cm^2^) and Brand F (7.13 kg/cm^2^) respectively. The disintegration time for ten brands of ciprofloxacin tablet obtained were in the subsequent order: Brand G (8.19 min) < Brand C (9.25 min) < Brand E (9.61 min) < Brand D (10.11 min) < Brand B (11.07 min) < Brand A (12.15 min) < Brand H (13.68 min) < Brand I (14.59 min) < Brand J (16.32 min) < Brand F (17.49 min). Among ten brands for dissolution test mean percentages of drug release were not less than 80% in 45 min for four tablets (Brand E, 81.52%; Brand D, 86.44%; Brand G, 86.82% and Brand C, 94.12%), consequently they met BP standard and as per USP-NF standard six brands (Brand B, 75.62%; Brand A, 76.18%; Brand E, 81.52%; Brand D, 86.44%; Brand G, 86.82% and Brand C, 94.12%) had released not less than 75% drugs, so they also complied with the standard. The percentages of the drug content of the ten brands of ciprofloxacin tablet were obtained in the following sequence: Brand H (96.84%) < Brand J (97.34%) < Brand D (98.15%) < Brand I (98.47%) < Brand E (99.37%) < Brand F (100.28%) < Brand B (100.38%) < Brand A (100.54%) < Brand G (101.39%) < Brand C (101.46%). All of the brands met the BP and USP-NF specifications for assay. First-order, Higuchi and Korsmeyer–Peppas kinetics model fit for all of the mentioned ten brands.

**Conclusion:**

The present study revealed that all of the leading brands of this tablet met the quality control parameters as per pharmacopoeial specifications except dissolution test for four brands (Brand J, Brand H, Brand I, and Brand F).

## Background

Ciprofloxacin is an antibiotic in a group of drugs called fluoroquinolones [1]. In 1981 it was discovered by Bayer, Germany. The Food and Drug Administration (FDA) approved this drug in 1987 for uses in the United States as first oral broad-spectrum antibiotic [2]. It is one of the most important medications needed in the basic health care system and available on the World Health Organization’s (WHO) list of essential medicines [3]. It is available as a generic medication and not very expensive [4, 5]. Generic drugs must be chemically and biopharmaceutically equivalent in comparison to the innovator drug. Quality parameters such as strength, purity, content uniformity, disintegration time (DT) and dissolution rate must be identical for pharmaceuticals that are chemically and biopharmaceutically equivalent [6]. Generic drugs are not only decreasing the health care costs [2] but also the quality of the drugs. The qualities of generic drugs are in doubt in case of poor, developing as well as industrialized countries.

There are a number of cases related to substandard and counterfeit drugs. Composition and ingredients of substandard drugs don’t meet the correct scientific specifications for these reasons they are ineffective and often dangerous to the patient. Counterfeit drugs may include products with the correct ingredients but fake packaging, with the wrong ingredients, without active ingredients or with insufficient active ingredients [7]. It is believed that the health hazard effects of counterfeit drugs are greater than substandard drugs [8]. Substandard and counterfeit drugs are a major cause of morbidity, mortality and loss of public confidence in drugs and health structures [9].

WHO has estimated that approximately 10% of the global pharmaceuticals market consists of counterfeit drugs, but this estimate increases to 25% in developing countries, and may exceed 50% in certain countries [10]. FDA estimates that up to 25% of the drugs consumed in poor countries are substandard or counterfeit [11]. China and India are recognized as the leading countries in the production of counterfeit drugs and bulk active ingredients used for counterfeiting worldwide [12]. Several studies showed that counterfeits of pharmaceuticals sourced in China and India were detected in 42 and 33 countries respectively [13]. Almuzaini et al. showed that prevalence of substandard or counterfeit medicines in Lao PDR, Tanzania, Cambodia and Uganda are 12.2–44.5%, followed by Indonesia, Nigeria, Cameroon 18–48% and in Myanmar, Cambodia, Lao PDR, Ghana, Kenya, Tanzania, Uganda, Madagascar, Mali, Mozambique, Zimbabwe 11–44% [14]. In 2009, 25 children were dying after taking paracetamol syrup due to the presence of poisonous diethylene glycol in Bangladesh [15, 16]. Substandard and counterfeit drugs are not only limited to poor and developing countries but also intensely noticeable in developed countries. In 2007–2008, due to the uses of adulterated blood thinner, heparin 149 Americans were dying that was legally imported. In 2012, contaminated steroids killed 11 people and sickened another 100 people in the US. In another case, vials of the cancer medicine, avastin were found to contain no active ingredients [17]. In a study of WHO found that 28% of antibiotic and 20–90% of antimalarial drugs were failed quality specifications [18].

Pharmaceuticals must satisfy certain standards to claim it to be a quality drug. The main criteria for the quality of any drug in dosage form are its safety, potency, efficacy, stability, patient acceptability and regulatory compliance [19]. To ensure the safety and efficacy of pharmaceutical products, the quality of pharmaceutical must be reliable and reproducible from batch to batch [20]. To ensure the requisite quality, drug manufacturers are required to test their products during and after manufacturing at various intervals during the shelf-life of the product [21]. WHO supports the practice of prescribing of generic medicines to reduce the cost of the health care system, but this should be supported with adequate evidence for the substitution of one brand for another [22]. Comparison of bioequivalence study between the generic products versus the innovator product is one of the major challenges and prime factors for a generic marketing authorization [23]. Several studies showed that switching from branded to generic medicine might result in changes of pharmacokinetics/pharmacodynamics profile, leading to subtherapeutic concentration or therapeutic failure and or adverse reactions [24]. It is very essential to do bioequivalence studies for generic products on account of any significant difference in the rate and extent by which the therapeutic ingredients become available at the site of drug action, administered under uniform conditions in an adequately designed study [25]. To identify bioavailability problems dissolution testing serves as an indicator [26]. Biopharmaceutically as well as chemically equivalent drug products must have the same quality, strength, purity, content uniformity, disintegration and dissolution rates [27]. In vitro quality control (QC) of pharmaceutical products is a fixed set of investigation started during production by in-process quality control tests and after production by finished product quality control tests as per official pharmacopoeias and different regulatory agencies. QC tests help in avoiding the confusion regarding safety, potency, efficacy and stability of pharmaceuticals [28].

The prevalence of substandard and or counterfeit medicines is significantly higher in poor and developing countries. As ciprofloxacin is widely used antibiotic in Bangladesh, the objective of this study was to assess the quality of different leading brands of ciprofloxacin hydrochloride 500 mg tablet formulation commercially available in the market of Bangladesh.

## Methods

### Drugs and chemicals

Ten commercially available leading brands of ciprofloxacin hydrochloride tablet each with a label claim 500 mg were purchased from the various retail pharmacies of Dhaka city in Bangladesh. Details information about the brands are shown in Table 1. The samples were blindly named as Brand A, Brand B, Brand C, Brand D, Brand E, Brand F, Brand G, Brand H, Brand I and Brand J. The standard ciprofloxacin hydrochloride powder equivalent to ciprofloxacin 200 mg was obtained from the Modern Pharmaceuticals Ltd, Dhaka, Bangladesh. Unless otherwise specified, all other chemicals were of analytical grade.

**Table 1:** Label information of ten leading brands of ciprofloxacin tablets

Brand name	Manufacturing country	Manufacturing date	Expiring date
Ciprocin 500	Bangladesh	September 2015	September 2017
Beuflox 500	Bangladesh	June 2015	May 2017
Neofloxacin 500	Bangladesh	August 2015	August 2018
Ciprox 500	Bangladesh	August 2015	August 2018
Quinox 500	Bangladesh	February 2015	February 2018
Flontin 500	Bangladesh	September 2015	September 2018
Floxabid 500	Bangladesh	May 2015	May 2018
Cipro A 500	Bangladesh	March 2015	March 2018
Ciprozid DS 500	Bangladesh	June 2015	June 2018
Rocipro 500	Bangladesh	April 2015	April 2018

### Instruments

Instruments used in this study were mortar, pestle, electronic balance (Model: D455007359, Shimadzu Corp.), Roche friabilator (Model: 902, Intech REV), Monsanto hardness tester (Model: Mht-20, Campbell Elec.), USP disintegration apparatus (Model: LTD-DV, Intech), USP dissolution apparatus (Model: VDA-6DR, Veego Instruments Cor.) and ultra violet (UV) spectrophotometer (Model: UV-1800, Shimadzu Corp.).

### In vitro quality control tests

#### Weight variation test

For this test according to the USP-NF weight variation test was run by weighting 20 tablets for each of the ten brands individually using an electronic balance, then calculating the average weights and comparing the individual tablet weights to the average. The difference in the two weights was used to calculate weight variation by using the following formula [19, 29, 30]:

$${\text{Weight variation}} = ({\text{I}}_{\text{w}}-{\text{A}}_{\text{w}} )/{\text{A}}_{\text{w}} \times 100\%$$

where, I_w_ = Individual weight of the tablet and A_w_ = Average weight of the tablet.

The tablet complies with the test if not more than 2 of the individual weights deviate from the average weight by more than the 5% [19, 29, 30].

#### Friability test

For this test Roche friabilator was used. Twenty tablets from each of the ten brands were weighed and placed in the friabilator and then operated at 25 rpm for 4 min. The tablets were then dedusted and weighed. The difference in the two weights was used to calculate friability by using the following formula [19, 31]:

$${\text{Friability}} = ({\text{I}}_{\text{w}} - {\text{F}}_{\text{w}} )/{\text{I}}_{\text{w}} \times 100\%$$

where, I_w_ = Total Initial weight of the tablets and F_w_ = Total final weight of the tablets.

The tablet complies with the test according to USP-NF if tablets loss less than 1% of their weight [19, 31].

#### Hardness test

For this test Monsanto hardness tester was used. Ten tablets were randomly selected from each of the ten brands and tested. This test measures the pressure required to break diametrically placed tablets by applying pressure with coiled spring [19, 32]. In-house acceptable limit for this test is 6 ± 2 kg/cm^2^.

#### Disintegration test

For this test USP disintegration apparatus was used. To test for DT, one tablet was placed in each tube for each brand and the basket rack was positioned in a 1000 ml vessel containing 900 ml of water maintained at 37 ± 2 °C, so that the tablets remained 2.5 cm below the surface of the liquid on their upward movement and descent not closer than 2.5 cm from the bottom of the beaker. A standard motor driven device was used to move the basket assembly containing the tablets up and down through a distance of 5–6 cm at a frequency of 28–32 cycles per minute. Perforated plastic discs were used to prevent the floating of tablets. The apparatus was operated for 30 min [19, 29].

To comply with the USP-NF standards, the tablets must disintegrate and all particles must pass through the 10-mesh screen within 30 min. If any residue remains, it must have a soft mass with no palpably firm core [25, 29].

#### Dissolution test

For this test USP dissolution apparatus was used. To test for dissolution, one tablet was placed in each vessel (6 vessels) for each brand, containing 900 ml of 0.1 M hydrochloric acid (HCl) as a dissolution medium maintained at 37 ± 0.5 °C. The rotational speed of the apparatus was held constant at 50 rpm. A sample of 5 ml was withdrawn at a fixed time intervals (15, 30, 45 and 60 min) and this was immediately replaced with the same volume of fresh test media [25, 29, 33].

The sample was filtered and 1 ml of filtrate was taken and diluted to 50 ml with distilled water. So the solution was 50 times diluted. The absorbance of the diluted filtrate was determined spectrophotometrically at the wavelength of 276 nm, using 0.1 M HCl as blank. The percentage of drug release at each interval was calculated by using standard ciprofloxacin. As per USP-NF tablets meet with this test if not less than 75% dissolves in 45 min. According to BP tablet comply with this test if not less than 80% dissolves in 45 min [19, 29, 33].

#### Assay test

Analysis of drug potency in tablets helps to determine the strength or content of drug in a dosage form. 100 mg of standard ciprofloxacin hydrochloride powder was weighed and dissolved in 10 ml of distilled water and diluted up to 100 ml to get 1000 µg/ml concentration of standard stock solution. From this stock solution 10 ml was taken to another 100 ml volumetric flask and diluted to get 100 µg/ml of drug concentration. Then, using this stock solution various other concentrations were prepared like 5, 10, 15, 20, 25 and 30 µg/ml. Absorbance values of these concentrations were measured at 276 nm by using UV spectrophotometer and standard graph was plotted by taking absorbance values on Y-axis and concentration values on X-axis. For this test tablets from each brand were crushed into fine powder and sufficient amount of powder was weighed so that the amount contains 100 mg of active ciprofloxacin and dissolved in 100 ml 0.1 M HCl and further dilution was made to obtain 100 µg/ml for each brand. Then 4 ml of each brand made up to 100 ml with 0.1 M HCl and the absorbance of each brand was taken at 276 nm against the blank [21].

The USP-NF specification is that the content of ciprofloxacin hydrochloride should not be less than 90% and not more than 110%, while BP specifies that the content should not be less than 95% and not more than 105% [29, 33].

### Drug release kinetics

To evaluate the kinetics of drug release from the tablets the results of in vitro drug release study of formulations were fitted with various kinetic equations like zero-order, first-order, Higuchi and Korsmeyer–Peppas model [34]. The equations of different release kinetics are given below:

$${\text{Zero-order kinetics:}}\;{\text{Q}}_{\text{t}} = {\text{Q}}_{0} + {\text{K}}_{0} {\text{t}}$$

$${\text{First-order kinetics:}}\;\log {\text{Q}}_{\text{t}} = \log {\text{Q}}_{0} + {\text{K}}_{1} {\text{t}}/2.303$$

$${\text{Higuchi kinetics: Q}}_{\text{t}} = {\text{K}}_{\text{h}} {\text{t}}^{1/2}$$

$${\text{Korsmeyer}}{-}{\text{Peppas kinetics: Q}}_{\text{t}} /{\text{Q}}_{ 0} = {\text{Kt}}^{\text{n}}$$

where, K_0_, K_1_ and K_h_ indicates zero-order, first-order and Higuchi rate constants respectively, Q_t_/Q_0_ means fraction of drug released at time t, K means rate constant and n means release exponent. The kinetics that gives high regression coefficient (R^2^) value is considered as the best fit model [34–36].

### Statistical analysis

All the results were expressed as mean ± SD. The results of dissolution test were analyzed by one-way analysis of variance (ANOVA) followed by Post Hoc t test. Microsoft Excel 2010 (Roselle, IL, USA) was used for the statistical and graphical evaluations. A probability of P < 0.05 was considered as significant.

## Results

**Fig. 1:**
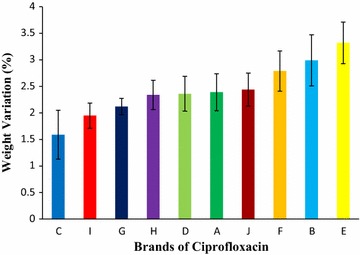
Results of weight variation test of ten leading brands of ciprofloxacin tablets. Results were expressed as mean ± SD (n = 20/brand)

The tablet complies with the weight variation test if not more than 2 of the individual weights deviate from the average weight by more than the 5 percent. The mean results for weight variation for ten brands obtained were in the following order: Brand C (1.59%) < Brand I (1.95%) < Brand G (2.12%) < Brand H (2.34%) < Brand D (2.36%) < Brand A (2.39%) < Brand J (2.44%) < Brand F (2.79%) < Brand B (2.99%) < Brand E (3.32%), given in Fig. 1. Among all tablets mean highest weight variation was found in Brand E, 3.32% and the lowest was found in Brand C, 1.59%. This means that all the brands complied with the compendial specifications.

**Fig. 2:**
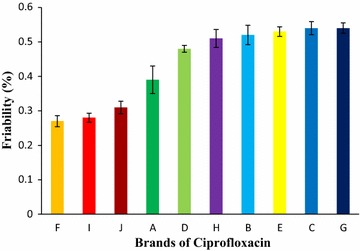
Results of friability test of ten leading brands of ciprofloxacin tablets. Results were expressed as mean ± SD (n = 20/brand)

Figure 2 showed the mean results of friability for ten brands of ciprofloxacin tablet in the subsequent order: Brand F (0.27%) < Brand I (0.28%) < Brand J (0.31%) < Brand A (0.39%) < Brand D (0.48%) < Brand H (0.51%) < Brand B (0.52%) < Brand E (0.53%) < Brand C (0.54%) < Brand G (0.54%). Thus, the brand most likely to lose particles during handling was Brand G, 0.54%, while the least likely to lose particles was Brand F, 0.27%. Friability for all the brands was below 1% and they complied with the compendial specifications.

**Fig. 3:**
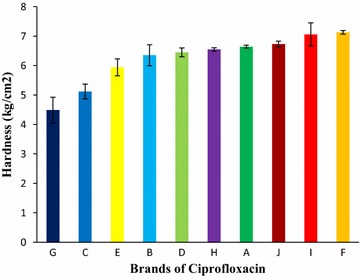
Results of hardness test of ten leading brands of ciprofloxacin tablets. Results were expressed as mean ± SD (n = 10/brand)

The mean hardness results (Fig. 3) for ten brands obtained were in the specified order: Brand G (4.49 kg/cm^2^) < Brand C (5.12 kg/cm^2^) < Brand E (5.94 kg/cm^2^) < Brand B (6.35 kg/cm^2^) < Brand D (6.45 kg/cm^2^) < Brand H (6.55 kg/cm^2^) < Brand A (6.64 kg/cm^2^) < Brand J (6.73 kg/cm^2^) < Brand I (7.06 kg/cm^2^) < Brand F (7.12 kg/cm^2^). From Fig. 3, it can be seen that Brand F, 7.12 kg/cm^2^ had the highest hardness value while Brand G, 4.49 kg/cm^2^ had the lowest value.

**Fig. 4:**
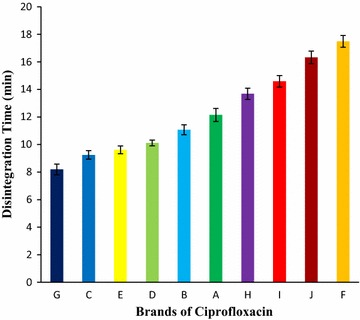
Results of disintegration test of ten leading brands of ciprofloxacin tablets. Results were expressed as mean ± SD (n = 6/brand)

Disintegration could be directly related to dissolution and subsequent bioavailability of a drug. The DT for ten brands of ciprofloxacin tablet obtained were in the succeeding order: Brand G (8.19 min) < Brand C (9.25 min) < Brand E (9.61 min) < Brand D (10.11 min) < Brand B (11.07 min) < Brand A (12.15 min) < Brand H (13.68 min) < Brand I (14.59 min) < Brand J (16.32 min) < Brand F (17.49 min). Highest DT was found in Brand F, 17.49 min and lowest was found in Brand G, 8.19 min. All the brands complied with the compendial specifications for this test given in Fig. 4.

**Fig. 5:**
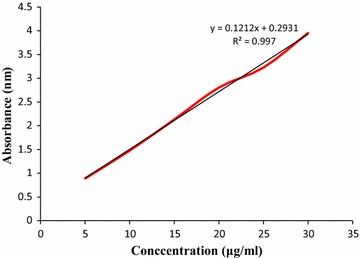
Standard calibration curve for ciprofloxacin hydrochloride

**Fig. 6:**
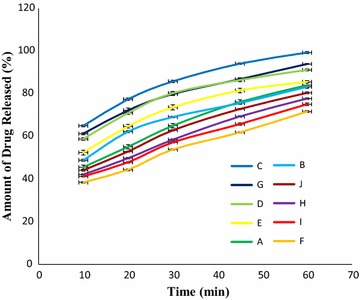
Results of dissolution profile of ten leading brands of ciprofloxacin tablets. Results were expressed as mean ± SD (n = 6/brand)

The calibration curve of standard ciprofloxacin is given in Fig. 5 (y = 0.1212x + 0.2931, R^2^ = 0.997). The percentages of drug release for ten brands of ciprofloxacin tablet in 45 min were in the resulting order: Brand F (61.87%) < Brand I (65.77%) < Brand H (69.36%) < Brand J (72.86%) < Brand B (75.62%) < Brand A (76.18%) < Brand E (81.52%) < Brand D (86.44%) < Brand G (86.82%) < Brand C (94.12%), shown in Fig. 6. According to BP the percentages of drug release at 45 min were less than 80% for Brand F, Brand I, Brand H, Brand J, Brand B and Brand A. But among these six brands Brand B and Brand A met the USP-NF standard.

**Fig. 7:**
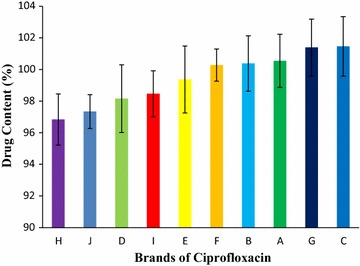
Results of drug content of ten leading brands of ciprofloxacin tablets. Results were expressed as mean ± SD (n = 20/brand)

The percentage of the drug per tablet was then measured using the calibration curve represented in Fig. 5 (y = 0.1212x + 0.2931, R^2^ = 0.997). The percentages of the drug content of the ten brands of ciprofloxacin tablet were obtained in the stated sequence: Brand H (96.84%) < Brand J (97.34%) < Brand D (98.15%) < Brand I (98.47%) < Brand E (99.37%) < Brand F (100.28%) < Brand B (100.38%) < Brand A (100.54%) < Brand G (101.39%) < Brand C (101.46%). All of the brands met the BP and USP-NF specifications (Fig. 7) for assay.

The statistical evaluation (ANOVA) of dissolution test given in Table 2 showed that there was significant variation (P < 0.05) found among ten brands of ciprofloxacin tablets. The kinetics of drug release of the proposed brands (Brand A, Brand B, Brand C, Brand D, Brand E, Brand F, Brand G, Brand H, Brand I and Brand J) were treated in different kinetics model such as zero-order, first-order, Higuchi and Korsmeyer–Peppas mentioned in Table 3.

**Table 2:** Results of ANOVA for dissolution test of ten leading brands of ciprofloxacin tablets

Source of variation	SS	df	MS	F	P value	F crit
Between groups	4522.771	9	502.5302	2.660375	0.016104	2.124029
Within groups	7555.779	40	188.8945			
Total	12078.55	49

**Table 3:** Kinetics of drug release from ten leading brands of ciprofloxacin tablets

Brands	Zero order kinetics	First order kinetics	Higuchi kinetics	Korsmeyer–Peppas kinetics
	Regression coefficient (R^2^)			
Brand A	0.920	0.943	0.974	0.992
Brand B	0.924	0.982	0.976	0.991
Brand C	0.913	0.974	0.969	0.988
Brand D	0.955	0.981	0.974	0.999
Brand E	0.943	0.983	0.981	0.989
Brand F	0.973	0.999	0.999	0.976
Brand G	0.981	0.983	0.998	0.989
Brand H	0.977	0.999	0.957	0.965
Brand I	0.961	0.973	0.973	0.985
Brand J	0.972	0.985	0.960	0.980

## Discussion

Quality is not an accident, it is the result of intelligent effort [37]. The quality of pharmaceuticals is under great risks in developing countries especially in Bangladesh. There are several factors related to bad quality among these uses of substandard raw material and lacks of facility are most prominent. Therefore, it is necessary to check the quality. Pharmacopeial testing confirms these properties according to fixed standards. Different brands of ciprofloxacin hydrochloride tablets were obtained from different retail pharmacy outlets within Dhaka City and were subjected to weight variation, friability, hardness, disintegration, dissolution and assay tests.

The weight of the tablet is the amount of granules which contains the labeled amount of the therapeutic ingredient. A large weight variation precludes good content uniformity. Due to a variety of reasons tablets may be excessively overweight or underweight. Patients receiving the overdose or underdose tablet, experiences unpredictable therapeutic response [38]. The tablet complies with this test if not more than 2 of the individual weights deviate from the average weight by more than the 5 percent. The mean results of weight variation for ten brands obtained were in the following order: Brand C (1.59%) < Brand I (1.95%) < Brand G (2.12%) < Brand H (2.34%) < Brand D (2.36%) < Brand A (2.39%) < Brand J (2.44%) < Brand F (2.79%) < Brand B (2.99%) < Brand E (3.32%) revealed in Fig. 1. Among ten brands of ciprofloxacin tablet, all of the brands met the specification, highest weight variation was seen in Brand E and the lowest was in Brand C. In the study of quality control and in vitro bioequivalence studies on four brands of ciprofloxacin tablets commonly sold in Uyo Metropolis, Nigeria, Jackson et al. also reported similar results [6].

Friability of tablet is the capacity to withstand shock and abrasion in packaging, handling and shipping. Friable tablets no longer have sharp edges, consequent pharmaceutical elegance and patient acceptance. Tablet friability results in weight loss of tablets which may affect the therapeutic response [39]. The mean results of friability for ten brands obtained were in the subsequent order: Brand F (0.27%) < Brand I (0.28%) < Brand J (0.31%) < Brand A (0.39%) < Brand D (0.48%) < Brand H (0.51%) < Brand B (0.52%) < Brand E (0.53%) < Brand C (0.54%) < Brand G (0.54%) exposed in Fig. 2. The results for friability were below 1% for ten brands of ciprofloxacin tablets, which met the specification. Salih and Hamam in the study of comparative in vitro evaluation of generic ciprofloxacin hydrochloride tablets showed that all the four different manufacturing brands of ciprofloxacin tablets complied with USP-NF requirements for this test [40].

Tablets require a certain amount of hardness to withstand mechanical shocks of handling in manufacturing, packaging and shipping. In addition, tablets should be able to withstand reasonable abuse when in the hands of consumers. Adequate tablet hardness and resistance to powdering are necessary requisites for consumer acceptance. More recently, this relationship of hardness to tablet disintegration and perhaps more significantly, to the drug dissolution release rate, has become apparent [41]. Tablet hardness can be attributed to the difference in properties of excipients employed in the manufacture of the different brands. Hardness values did not correlate with friability values [42]. The mean results of hardness for ten brands obtained were in the resulting order: Brand G (4.49 kg/cm^2^) < Brand C (5.12 kg/cm^2^) < Brand E (5.94 kg/cm^2^) < Brand B (6.35 kg/cm^2^) < Brand D (6.45 kg/cm^2^) < Brand H (6.55 kg/cm^2^) < Brand A (6.64 kg/cm^2^) < Brand J (6.73 kg/cm^2^) < Brand I (7.06 kg/cm^2^) < Brand F (7.12 kg/cm^2^) exhibited in Fig. 3. But the Figs. 2 and 3 showed that highest friable brand, Brand G has lowest hardness and lowest friable brand, Brand F has highest hardness. All of the tablets met the in-house specification for this test. In the bioequivalence studies on some selected brands of ciprofloxacin hydrochloride tablets in the Nigerian market with ciproflox^®^ as innovator brand Ayodeji et al. showed that all of the brands comply with the specification for this test [43].

Before absorption of drug takes place in the body, it must be in solution form. For most tablets the first important step toward solution is the breakdown of the tablet into smaller particles or granules, a process known as disintegration [44]. Disintegration must be directly related to dissolution and subsequent bioavailability of a drug [45]. The DTs for ten brands were under 30 min. The mean results of DT for ten brands obtained were in the aforementioned order: Brand G (8.19 min) < Brand C (9.25 min) < Brand E (9.61 min) < Brand D (10.11 min) < Brand B (11.07 min) < Brand A (12.15 min) < Brand H (13.68 min) < Brand I (14.59 min) < Brand J (16.32 min) < Brand F (17.49 min) shown in Fig. 4. As per results shown, Brand G has lowest DT and Brand F has highest DT, but all of the brands meet the compendial requirements. Similar findings were reported by Kahsay and Egziabher [46].

When a drug is administered orally in the form of the tablet, the absorption of the tablet depends on how fast it goes into solution, i.e., absorption of a drug is totally dependents on the dissolution of the tablet. Dissolution is a rate limiting step prior to absorption. The rate of dissolution is directly related to the efficacy of the tablet products, as well as to bioavailability difference between formulations [47]. To meet the BP standard the percentages of drug release at 45 min must be not less than 80 and 75% according to USP-NF standard. The percentages of drug release for ten brands of ciprofloxacin tablet in 45 min were in the succeeding order: Brand F (61.87%) < Brand I (65.77%) < Brand H (69.36%) < Brand J (72.86%) < Brand B (75.62%) < Brand A (76.18%) < Brand E (81.52%) < Brand D (86.44%) < Brand G (86.82%) < Brand C (94.12%), presented in Fig. 6. Among ten brands the percentages of drug release were more than 80% for four brands (Brand E, Brand D, Brand G and Brand C) and less than 80% for six brands (Brand F, Brand I, Brand H, Brand J, Brand B and Brand A) as per BP standard. But according to USP-NF standard six brands (Brand B, Brand A, Brand E, Brand D, Brand G and Brand C) complied with the specification and remaining four brands (Brand F, Brand I, Brand H and Brand J) did not comply with the specification given in Fig. 6. In the study on in vitro quality assessment and bioequivalence studies on four brands of ciprofloxacin tablets, marketed in Ambo, Ethiopia, Fereja et al. stated that out of four brands of ciprofloxacin tablets one brand failed to meet the dissolution profile [48].

Analysis of the assay of the drug is very important to determine the presence, absence, or quantity of one or more components in the dosage form [19]. In this study ciprofloxacin hydrochloride tablets were assayed by using UV spectroscopic technique due to lack of instrumentation. A number of literatures suggest the UV spectroscopy for the analysis of ciprofloxacin hydrochloride tablet [49]. In fact there is no major problem in the assay of ciprofloxacin tablet by UV spectroscopic technique instead of high performance liquid chromatography technique. The percentages of the drug content of the ten brands of ciprofloxacin tablet were obtained in the following sequence: Brand H (96.84%) < Brand J (97.34%) < Brand D (98.15%) < Brand I (98.47%) < Brand E (99.37%) < Brand F (100.28%) < Brand B (100.38%) < Brand A (100.54%) < Brand G (101.39%) < Brand C (101.46%) displayed in Fig. 7. All of the brands were complied with the BP and USP-NF specification for assay test. The highest percentage of drug content was obtained for Brand C (101.46%), whereas the lowest percentage of drug content was obtained from Brand H (96.84%) given in Fig. 7. Usman et al., in the evaluation of dissolution testing for ciprofloxacin (500 mg) tablets: post market surveillance of different brands available in Ras Al Khaimah (UAE) showed that all ciprofloxacin tablets comply with the content assay test [34].

To compare the quality of all the ten brands used in the study ANOVA was performed. Results presented in Table 2 indicate that there are no significant differences in the release pattern of different brands at P < 0.05 and the F value (2.660375) is higher than F crit value (2.124029). Table 3 shows the different kinetics model that was used to plot various parameters for considering the determination of R^2^. It shows that the all of the mentioned kinetics fit for all brands. However the first-order kinetics, described the drug dissolution with R^2^ approximately 1 for Brand F and Brand H, Higuchi kinetics model for Brand F and Brand G and Korsmeyer–Peppas kinetics model for Brand A, Brand B and Brand D respectively. Zero-order kinetics did not best fit to any brands. Among ten brands only Brand F and Brand H were best fit model for first-order kinetics. In case of Brand F and Brand G, Higuchi kinetics model was the best fit model. Korsmeyer–Peppas kinetics model was the best fit model for Brand A, Brand B and Brand D among ten brands.

## Conclusion

From the present study it was clearly demonstrated that all of the leading brands of the ciprofloxacin hydrochloride tablet met the criteria laid in the official monographs for in vitro quality control tests except dissolution test for four brands (Brand F, Brand I, Brand H and Brand J). But each brand should meet dissolution criteria to be therapeutically effective. So the healthcare professionals should focus on quality rather gift items to ensure better health of people. It will ultimately force the pharmaceutical industry to invest more in quality to ensure better pharmaceuticals for the betterment of the health sector of the country.
